# Community Pharmacists’ Contributions to Disease Management During the COVID-19 Pandemic

**DOI:** 10.5888/pcd17.200317

**Published:** 2020-07-23

**Authors:** Mark A. Strand, Jeffrey Bratberg, Heidi Eukel, Mark Hardy, Christopher Williams

**Affiliations:** 1 Professor, School of Pharmacy and Department of Public Health, North Dakota State University, Fargo, North Dakota; 2 Clinical Professor, College of Pharmacy, The University of Rhode Island, Kingston, Rhode Island; 3 Executive Director, North Dakota State Board of Pharmacy, Bismarck, North Dakota; 4 Associate Professor of Pharmacology, Division of Pharmaceutical Sciences, Xavier University of Louisiana, New Orleans, Louisiana

## Abstract

Community pharmacists assist patients to manage disease and prevent complications. Despite the enormous challenge the coronavirus disease 2019 (COVID-19) pandemic has dealt to the health care system, community pharmacists have maintained the delivery of critical health services to communities, including those most at risk for COVID-19. Community pharmacists are in a key position to deliver priority pandemic responses including point-of-care testing for chronic disease management, vaccinations, and COVID-19 testing.

**Summary**

**What is already known on this topic?**

More than 90% of people in the United States live within 5 miles of a community pharmacy. Pharmacists deliver important public health services such as vaccinations, point-of-care testing, and chronic and acute disease prevention and management. These services are and will continue to be critical in the coronavirus disease 2019 (COVID-19) pandemic.

**What is added by this report?**

The COVID-19 pandemic has demonstrated needed roles for the community pharmacist in an emergency, including continuity of provision of medications, providing preventive services, and ensuring health equity. Along with medication management, pharmacists provide infectious disease mitigation, point-of-care testing, and vaccinations.

**What are the implications for public health practice?**

Community pharmacists are essential contributors to public health and play a key role as the United States continues to combat COVID-19, especially among populations with health disparities.

## Background

The coronavirus disease 2019 (COVID-19) pandemic has challenged community pharmacists to perform under difficult circumstances. The pandemic has also highlighted the key public health functions community pharmacists play in medication therapy, chronic disease management, self-care recommendations, vaccinations, point-of-care screening and testing services, and adherence support (1–4). Although the role of pharmacists in chronic disease prevention and management is well established, the COVID-19 pandemic has accentuated the critical contributions community pharmacists make during an infectious disease outbreak.

This commentary describes the current and future roles of community pharmacists in the United States in optimizing their broad access to medically and socially vulnerable populations before and during a pandemic. We show that community pharmacists are highly accessible both temporally and geographically, which puts them in a position to serve at-risk populations. The ongoing role of community pharmacists in preventing and managing common diseases during a pandemic is also addressed. Finally, we describe the key roles pharmacists play in priority pandemic responses, including point-of-care testing for chronic disease management, testing for COVID-19, and administering and advocating for vaccinations.

## Community Pharmacists in the United States

Community pharmacies are located in most communities in the United States, and more than 90% of the US population live within 5 miles of one (5). Furthermore, patients visit their community pharmacist 12 times more frequently than their primary care provider (6). As medication experts, community pharmacists fill a key role in providing care for patients with chronic diseases (Table 1), with particular contributions made among economically and geographically underserved populations (8). When many health care organizations restricted patient access to noncritical services in the early stages of the COVID-19 pandemic, patients with chronic diseases struggled to receive routine care. Through the thoughtful implementation of social distancing guidelines, most pharmacies remained open and were in a position to support patients (9). These critical services included medication dispensing for chronic and acute conditions, vaccinations, recommendations for over-the-counter medications, and medication management (10).

**Table 1:** Pharmacist Interventions and Anticipated Outcomes in Contributing to Population Health^a^

Intervention	Anticipated Outcomes
**Prevention**	
Medication monitoring	Provide appropriate preventive medications Address medication access issues in the face of pandemic restrictions
Patient education	Educate patients about preventing coronavirus disease 2019 (COVID-19) infection and symptoms of the disease Provide education on over-the-counter medications Increase patient self-efficacy and reduce adverse outcomes from medications
Vaccinations	Reduce novel severe acute respiratory syndrome coronavirus 2 (SARS-CoV-2) transmission when a vaccine becomes available Prevent outbreaks of vaccine-preventable diseases
Point-of-care testing	Increase access to COVID-19 testing and reduce transmission by early detection and quarantine of detected individuals
**Management**	
Medication monitoring	Increase treatment success
Patient education	Educate patients about COVID-19 disease Increase patient self-efficacy and reduce adverse outcomes from medications
Medication therapy review	Optimize patient medication adherence and quality of life
Disease self-care and support	Ensure access when medical facilities are not accepting patients Empower patients, increase pharmacist role in multidisciplinary team, and improve population health
Point-of-care testing	Provide real-time point of care screening results for chronic disease management

a Based on Greer N, Bolduc J, Geurkink E, Rector T, Olson K, Koeller E, et al. Pharmacist-led chronic disease management: a systematic review of effectiveness and harms compared with usual care (7).

The COVID-19 pandemic has resulted in an excessive burden of mortality among at-risk populations, a burden exacerbated by pre-existing racial and socioeconomic inequities in health care access and use (11–14). The proportion of COVID-19 deaths among Black and American Indian/Alaska Native people is in excess of their weighted population distributions compared with other racial/ethnic groups (Table 2). Hypertension, diabetes, and respiratory diseases are disproportionately prevalent among communities of color (16), resulting in exponentially higher mortality among minority populations than among White populations (17). COVID-19 has brought into full view the need to address health inequities experienced by some segments of the US population (18).

**Table 2:** Comparison of Proportion of US Deaths From Coronavirus Disease 2019 (COVID-19) and Weighted Population Distribution by Race/Ethnicity^a^

Race/Ethnicity	Percentage of US Population	Percentage of COVID-19 Deaths	States With Known Racial Disparity in Outcomes
Asian	10.7	5.0	Nevada
Black	17.2	23.0	Alabama, District of Columbia, Georgia, Illinois, Kansas, Louisiana, Maryland, Michigan, Mississippi, Missouri, New York, South Carolina, Texas, Wisconsin
Hispanic or Latino	16.6	27.7	None
American Indian/Alaska Native	0.3	0.7	Arkansas, New Mexico, Oklahoma
Non-Hispanic White	42.3	53.4	Florida, Indiana, Kentucky, Massachusetts, Minnesota, New Hampshire, New Jersey, Ohio, Oregon, Pennsylvania, Rhode Island, Tennessee, Washington

a Table modified from Centers for Disease Control and Prevention, Weekly updates by select demographic and geographic characteristics, June 24, 2020, Table 2a (15).

Community pharmacies have opportunities to redress racial and ethnic disparities in health care delivery because of their accessibility (8). Pharmacies are located close to at-risk populations, such as in rural areas or areas with higher concentrations of people of lower socioeconomic status (19). During the pandemic, pharmacists have been able to leverage their social capital with their patients in those areas, and safely maintain patient access to essential medications through curbside pickup, larger refill quantities, and home delivery (20,21). Through close partnerships with pharmacy associations, corporate and individual ownership networks, and providers, pharmacists prepared for and have met the need for surges of chronic disease medication prescriptions and for potentially beneficial COVID-19 therapies (22). These actions have shown that community pharmacies are key players in addressing the pandemic and in ensuring health equity among patients.

Others at disproportionate risk of COVID-19 are people aged 60 or older, health care workers, and medically vulnerable patients with underlying chronic diseases (23). When these people develop severe COVID-19, they are hospitalized more frequently and die at higher rates (24,25). This is particularly true of patients with diabetes, cardiovascular disease, hypertension, chronic obstructive pulmonary disease, chronic kidney disease, and possibly pregnant women (23,26). Community pharmacists play a significant role in caring for patients with these conditions because these patients are frequently on chronic medications. Therefore, community pharmacists are in a position to educate patients about the importance of protecting themselves from exposure to COVID-19.

Concerns about health equity have been raised as the COVID-19 pandemic continues to change the landscape of public health and health care delivery (13,27). All aspects of health care need to be reevaluated with regard to how they may contribute to reducing inequality and increasing health equity. The role that community pharmacists play in providing care for at-risk populations must be included in this evaluation.

## Community Pharmacists’ Response During COVID-19 Pandemic 

Community pharmacies have continued to deliver critical services to their patients during the COVID-19 pandemic (10). In support of these efforts, the Centers for Disease Control and Prevention provided substantial guidance for pharmacists to ensure the safety of their workforce and their patients while simultaneously ensuring uninterrupted patient care (20). Two key roles played by community pharmacists are point-of-care testing and vaccinations.

### Point-of-Care Testing

In the absence of proven treatment medications or vaccines to prevent transmission, the priority actions to protect the public against COVID-19 and to mitigate future waves of infection are to test, trace, and quarantine people who are infected or exposed. These roles are assumed by local public health services; however, community pharmacists can play a significant role in COVID-19 testing (28). More than 10,000 pharmacies already perform Clinical Laboratory Improvement Amendments (CLIA)-waived tests to detect influenza and streptococcal pharyngitis and to monitor chronic diseases through a wide range of CLIA-waived point-of-care testing, such as finger stick glucose, Hb_A1c_, lipid panel, and more. These tests provide pharmacists with objective data in real time to educate patients about results, lifestyle recommendations, and referral to care. Therefore many pharmacies are authorized and prepared to incorporate COVID-19 testing into their workflow.

The COVID-19 pandemic has changed the landscape of primary care. Many patients have consulted health care providers via telehealth or cancelled their preventive care appointments (29), and these practices may continue for some time. Globally, COVID-19 has substantially affected services for noncommunicable diseases (30), which may leave a gap in chronic disease management, with people missing needed laboratory tests such as blood glucose, Hb_A1c_, or lipid screening (7). This screening gap is an area that awaits evaluation as the consequences of the COVID-19 pandemic become clearer. Because people who postpone screening will continue to receive their medications from their pharmacies, community pharmacists will have the opportunity to encourage patients to receive these screenings to ensure effective chronic disease management.

In addition to point-of-care testing for chronic disease management, pharmacists will also play a key role in COVID-19 testing (31). Pharmacists across the country have been called on to coordinate the administration of COVID-19 tests (32–34). In the future, providing ongoing COVID-19 surveillance to communities by allowing walk-in testing at community pharmacies might be more sustainable and convenient than the large-scale public screening being done as of the summer of 2020. By the fall of 2020, many pharmacies will be offering 1 or more of the following COVID-19 diagnostic services: selling home testing kits, collecting specimens to send to partner laboratories for testing and reporting, collecting specimens for on-site symptomatic testing and reporting, and collecting specimens for point-of-care antibody surveillance (31,35,36). The US Department of Health and Human Services has authorized all pharmacists to provide these COVID-19 testing services, overriding state law where it exists (37). The Centers for Medicare and Medicaid Services (CMS) is reimbursing pharmacies for this COVID-19 testing, overcoming a major hurdle to pharmacy-based clinical and diagnostic services during the pandemic (38).

### Vaccinations

Community pharmacists play a key role in advocating for and administering adult vaccines (39) (Table 1). Pharmacists must work to provide essential vaccinations to everyone entrusted to their care, especially children and at-risk populations who have fallen behind because of medical office closures (40). Additionally, community pharmacists will be key players in wide-scale administration of vaccines once a safe vaccine for the novel severe acute respiratory syndrome coronavirus 2 (SARS-CoV-2) is available. This will make vaccines widely available in convenient locations and in familiar settings. Now is the time for community pharmacy organizations to prepare for this critical public health role. Additionally, the community pharmacist’s role in providing accurate health information about COVID-19 and the safety and appropriateness of vaccines will continue (41).

## Implications for Public Health

In addition to ensuring uninterrupted delivery of routine pharmacy services, pharmacists are able to respond quickly to fill public health roles during a pandemic. Pharmacists have other opportunities to contribute even further to delivering upstream preventive health care measures while mitigating social and structural determinants of health in underserved and marginalized communities. Pharmacy-based community clinics, led by public health pharmacists and primary care providers, may become a common feature in community pharmacies. Pharmacist-provided vaccinations, specimen collection, and point-of-care testing will establish rapid and convenient diagnosis and surveillance of both acute and chronic diseases. Because a pharmacy is likely to be located in or near acute or chronic disease hotspots, and have real-time communication links to public health and primary care authorities, pharmacists can help public health leaders detect and prepare for surges of known and novel diseases. However, this will require deeper integration of pharmacy with the public health infrastructure than currently exists, a clear opportunity for future growth.

The United States has been hit particularly hard by the COVID-19 pandemic, revealing significant and widespread vulnerabilities and structural health disparities that challenge its health care system. The slow and uneven responses to COVID-19 indicate a public health infrastructure that lacks the resources and the authority to tackle such challenges. One reason is the lack of sustained resources to build strong public health infrastructures at the state, county, and city levels across the country (42). Furthermore, although progress has been made, the interfacing of public health in the United States with other sectors of the health care system, including community pharmacy, need to be strengthened to better prepare for quick response to a public health crisis (43). Twelve leading pharmacy organizations have signed the Pharmacy Organization’s Joint Policy Recommendations to Combat the COVID-19 Pandemic to delineate key roles pharmacists play in the response (31). Among the recommendations are authority to test, treat, and vaccinate patients; easing operational barriers to address workforce issues; addressing drug shortages; reimbursement for services provided; and removal of barriers to reimbursement. These all represent growth opportunities for collaboration between public health and pharmacy.

During this pandemic, and in past pandemics, the importance of community pharmacies and pharmacists in public health and the health of their patients has been evident (10). It is imperative that systematic evaluation and dissemination of pharmacists’ contributions be undertaken to determine areas where community pharmacy can best be incorporated into the way public health is operationalized and carried out in the United States. The COVID-19 pandemic has created the opportunity to strengthen the US public health system to make it even more inclusive, accessible, and effective.

The COVID-19 pandemic has challenged health care systems all over the world. During this pandemic, the community pharmacist has provided critical health services to communities, including those most at risk for COVID-19. As the role of the community pharmacist during the COVID-19 pandemic continues to evolve, pharmacy’s impact on improving patient and population health outcomes should be evaluated. The COVID-19 pandemic will likely reveal new roles that community pharmacists can play during a pandemic and beyond.
