# Erratum, Volume 17, July 23, 2020 Release

**DOI:** 10.5888/pcd17.200317e

**Published:** 2020-09-03

In Table 2 of the article “Community Pharmacists’ Contributions to Disease Management During the COVID-19 Pandemic,” data for the row labeled “Hispanic or Latino” were reversed. That row should read Percentage of US Population, 16.6; Percentage of COVID-19 Deaths, 27.7. The article was corrected on July 30, 2020, and appears online at https://www.cdc.gov/pcd/issues/2020/20_0317.htm. We regret any confusion or inconvenience this error may have caused.

